# Change in Health-Related Quality of Life among Pulmonary Tuberculosis Patients at Primary Health Care Settings in South Africa: A Prospective Cohort Study

**DOI:** 10.1371/journal.pone.0151892

**Published:** 2016-05-03

**Authors:** Julia S. Louw, Musawenkosi Mabaso, Karl Peltzer

**Affiliations:** 1 HIV/STI and TB (HAST) Research Programme, Human Sciences Research Council, South Africa; 2 Department of Research & Innovation, University of Limpopo, Sovenga, South Africa; 3 ASEAN Institute for Health Development, Madidol University, Salaya, Phutthamonthon, Nakhonpathom, Thailand; University of Malaya, MALAYSIA

## Abstract

**Introduction:**

Pulmonary tuberculosis (TB) remains a major public health challenge in South Africa. However, little attention is paid to the impact of health related quality of life (HRQL) among TB patients at the beginning and at the end of TB treatment. This study assesses factors associated with HRQL among tuberculosis patients in three high risk provinces in South Africa.

**Methods:**

A prospective cohort study was conducted at primary health care settings. Patients completed the HRQL social functioning (SF)-12 health survey. Comparison of Physical Health Summary Score (PCS) and Mental Health Summary Score (MCS) was assessed at 6 months after treatment. Generalized estimating equations (GEEs) were used to examine the factors associated with changes in HRQL.

**Results:**

In all patients, after 6 months of treatment there was a significant improvement in HRQL with the biggest increase in the PCS. A higher educational qualification had a strong significant positive effect on the mental HRQL. Psychological distress showed a significant negative association with physical and mental HRQL after six months. Permanent residence showed a significant positive association with mental HRQL among TB patients compared to those living in shack/traditional dwellings. Rating ones health as being good and fair/poor was significantly associated with poor physical HRQL. Twenty drinks or more in the past month had a significant negative effect on the physical HRQL.

**Conclusion:**

The findings suggest that programmes targeted at improving TB treatment success should have specific interventions for patients with low educational background, impoverished households/communities and those with hazardous or harmful alcohol use.

## Introduction

Tuberculosis (TB) remains a major public health challenge in South Africa, the country is ranked 6^th^ globally after India, Indonesia, China, Nigeria and Pakistan with an estimated 450,000 incident cases of active TB (ranging from 410 000−520 000) reported in 2014 [1, 2]. Furthermore, the rate of TB is on the rise and to add to the challenge, the TB epidemic is fuelled by high HIV co-infection rates and compounded by drug resistant forms of the disease [3]. Consequently, TB notification in South Africa has increased over the last 20 years [3] with the number of cases detected for all forms of TB steadily increasing each year [1]. As such, patients' health related quality of life (HRQL) may be diminished by the disease, side effects from medication and in some cases prolonged treatment duration [4]. However, the effect of TB disease on quality of life (QoL) over time is seldom considered and studied [5] because considerable attention has focused on preventing transmission and treatment outcome.

In clinical research, health related quality of life (HRQL) has become an accepted outcome measure [6] and it has been described as an individual’s own perception of well-being in physical, psychological and social aspects [7]. Physical and mental distress is found to be common in TB patients and as a result leading to poor disease outcome or poor treatment outcome [8]. Physical functioning reflects the capacity of the patient to carry out basic day-to-day activities, while psychological health takes into account several facets of the individual's mood and emotional well-being [9].The disease also moderately affects the daily activities of nearly half of the patients with tuberculosis, and most patients are worried, frustrated, or disappointed by the diagnosis, and almost a quarter do not initially accept their diagnosis [9].

A few international studies measured HRQL among active TB patients at the start, middle, and end of treatment [10, 11, 12]. Both Chamla [10] and Marra et al. [12] found significant improvement in all physical health subscales using the Short Form Health Survey (SF-36) after the anti-TB treatment. Kruijshaar et al. [13] followed patients at diagnoses and at 2 months using SF-36 and found small improvement in Physical Component Summary (PCS) score but a greater improvement in Mental Component Summary (MCS) score. Deribew et al [14] compared change in QoL in patients with HIV infection with and without TB and found severe common mental disorder to be strongly associated with poor HRQL among TB/HIV co-infected patients. Similarly Atif et al [15] found in their prospective study of HRQL that patients were at the risk of depression at the end of their TB treatment. HRQL as reported by patients, is therefore highly relevant to understanding the effect of TB treatment and associated factors. In SA, few studies have been conducted among TB patients specifically on HRQL and very few compared factors associated with HRQL at the beginning of TB treatment and at the end of TB treatment [3, 16].

The purpose of the current study was to assess factors associated with HRQL using the social functioning (SF-12) Health Survey among pulmonary tuberculosis patients at the beginning of treatment and six months following completion of treatment at primary health care settings in three high risk provinces in South Africa.

## Methods

### Study setting

The study included KwaZulu-Natal, Eastern Cape and Northern Cape, three provinces with the highest TB caseloads in South Africa [17]). These are the provinces that report the highest proportions of TB deaths in the country [18]. One district in each of these 3 provinces included Siyanda in Northern Cape Province, Nelson Mandela Metro in the Eastern Cape Province and eThekwini in KwaZulu-Natal Province. Within each of these three study districts 14 primary health care facilities were selected on the basis of the highest TB caseloads per clinic (N = 40).

### Study design and participants

A prospective cohort study was conducted among pulmonary TB patients with hazardous or harmful alcohol use in public primary care clinics to assess the impact of screening and brief interventions for hazardous and harmful alcohol use treatment at baseline and 6 months after completion of treatment described in detail elsewhere [17]. Both male and female new TB treatment and retreatment patients (within one month of TB treatment) 18 years or older that were screened positive for hazardous or harmful alcohol use based on the AUDIT score questionnaire were included in the study. Hazardous or harmful alcohol use was based on the AUDIT classification as hazardous or harmful alcohol use. All the participants’ in this study were taking anti-TB treatment within the DOTS programme which required them to attend the clinic during the week and take their anti-TB drugs dose at their homes over the week-end. A health care provider who identified a new TB treatment or retreatment patient 18 years and above informed the patient about the study and referred the patient for participation if they were willing. For all consenting patients a screening interview was conducted by trained research assistants. All patients who met the inclusion criteria and were willing to participate in the HRQL survey were included at baseline and followed up for 6 months.

### Ethical consideration

Ethical approval was obtained from the Human Sciences Research Council Research Ethics Committee (Protocol REC No.1/16/02/11). National, provincial and district health authorities also provided approval for this study. Written informed consent was obtained from the study participants. The data collection was anonymised to ensure confidentiality.

## Measurement Instruments

### Socio-demographic characteristics

A researcher-designed questionnaire was used to record information on participants’ age, sex, educational level, marital status, household income, dwelling characteristics and poverty index (availability of 5 essential items including shelter, fuel or electricity, clean water, food and cash income) at baseline.

### Health outcomes

The health-related behaviors that were measured included perceived health status, chronic conditions, psychological distress, smoking status, and level of alcohol consumption. Perceived health status was measured by participants rating their health on a 5-likert scale (excellent, very good, good, fair, poor). Chronic conditions were measured by participants indicating the number of chronic illnesses (including hypertension, diabetes, depression, stomach ulcer, cancer, etc.) that have been diagnosed in the past month. The Kessler Psychological Distress Scale (K10) was used to measure psychological distress [19]. The K10 scale consists of 5-point Likert items (1 = never, 2 = rarely, 3 = some of the time, 4 = most of the time, 5 = all of the time) to questions of how respondents felt during the previous 30 days. The 10 responses are added up and the total score is grouped into four categories representing likely to be well (score below 20), mild (score 20–24), moderate (score 25–29) and severe mental disorder (score 30 and above) [20]. Smoking status was determined by the participants’ self-report of whether they (a) use one or more of the following tobacco products (cigarettes, snuff, chewing tobacco, cigars), and (b) how often have they used one or more of the tobacco products? The level of alcohol consumption was assessed using the 10-item Alcohol Disorder Identification Test (AUDIT) [21]. Higher AUDIT scores (20 or more) indicate more severe levels of risk.

### Health related QoL measures

Health related QoL outcomes were assessed at baseline and 6 months using a validated instrument the social functioning (SF)-12 health survey which is a measure of general health functioning. The 12 items reflect eight sub-domains: self-perceived general health (1 item); bodily pain (1 item); physical functioning (2 items); physical role (2 items); vitality (1 item); general health (1 item); social functioning (1 item); mental health (2 items) and emotional role (2 items); Cronbach alpha at baseline is 0.67 and at 6-month follow-up 0.75.

For each respondent, the SF-12 scoring algorithm generates a Physical Component Summary (PCS-12) score and a Mental Component Summary (MCS-12) score. These scores are created by weighting and then summing the SF-12 item responses using two separate sets of weights (a physical weight and a mental weight) and then by normalizing the weighted sums to be comparable with a population mean score of 50 with a standard deviation of 10 [22]. Higher physical or mental SF-12 summary scores indicated better HRQL.

### Statistical analysis

Socio-demographic and health related characteristics were described using frequencies for categorical variables, means and standard deviation for continuous variables. Differences in patient characteristics at baseline and at 6 months were assessed using Chi-squared tests for categorical variables and paired t-tests for continuous variables. Generalized estimating equation (GEE) models with exchangeable working correlated structure were constructed [23] for the mental and physical scores to examine the factors associated with changes in HRQL. Time was included as a categorical variable in the models using the baseline, 3 and 6 month time points. Socio-demographic, socio-economic status, and health related variables were used as explanatory variables in addition to the treatment effect. A p-value ≤ 0.05 was considered statistically significant. Analyses were perfomed using STATA version 12.0 (Stata Corp., College Station, Texas).

## Results

Of the 4882 tuberculosis patients who screened for hazardous or harmful alcohol use and agreed to participate in the study 1,196 patients tested positive for the AUDIT and were willing to participate in the HRQL survey at baseline but only 853 (71.3%) participants were followed up at 6 months. The only statistically significant differences observed in socio-demographic characteristics due to 343 (28.7%) patients lost to follow up were in the significant reduction in the proportion of respondents by education level, poverty index, household income and dwelling type (Table 1). This was mainly due to misplaced fieldworker codebooks, incorrect recording of names that could not be matched with the clinic register and misplaced clinic registers at the clinic.

**Table 1:** Socio-demographics of the study sample at baseline and six months follow up.

Variables	Baseline	Follow-up (6months)		
	n = 1196	n = 853		
	n(%)	n(%)	chi2	p-value
Age in years (range 18–93)*				
18–24	126(10.7)	47(7.9)	8.429	0.134
25–34	438 (37.2)	218(36.6)		
35–44	329 (27.9)	159 (26.7)		
45–54	199(16.9)	114(19.1)		
55–64	70(5.9)	44(7.3)		
65 or more	14(1.1)	13(2.1)		
Gender*				
Female	331(27.9)	236(27.9)	0.001	0.986
Male	854 (72.0)	610(72.1)		
Marital status*				
Never married	816(72.2)	424(71.7)	12.1	0.06
Married/ cohabitating Separated/divorced/widowed	247(21.8)	137(23.1)		
	67(5.9)	27(4.5)		
Education*				
Grade 7 or less	384(32.6)	164(26.7)	14.81	<0.001
Grade 8–11	568(48.2)	354(57.7)		
Grade 12 or more	227(19.3)	95(15.5)		
Poverty index (5–20)*				
Low (5)	325(29.3)	223(37.2)	65.26	<0.001
Medium (6–12	542(48.9)	174(29.0)		
High (13–20)	242(21.8)	202(33.7)		
Household income*				
Formal salary	249(24.3)	115(19.4)	35.39	<0.001
Family contributions	392(38.2)	284(48.0)		
Social grants	213(20.8)	100(16.9)		
No income	171(16.7)	91(15.4)		
Dwelling type *				
Shack/traditional	389(35.4)	180(29.8)	13.07	0.011
Permanent	711(64.6)	417(69.1)		

* Totals do not add to overall total due to missing data

As highlighted in Table 2 below, there was a statistically significant improvement in all health related variables at six months follow up compared to baseline.

**Table 2:** Health related measures after six months follow up after treatment.

Variables	Baseline	Follow-up (6months)		
	n = 1196	n = 853		
	n(%)	n(%)	chi2	p-value
**Health variables**				
Perceived health status*				
Excellent/very good	227(19.2)	389(48.3)	223.68	<0.001
Good	416(35.1)	256(31.8)		
Fair/poor	541(45.7)	160(19.8)		
Chronic conditions*				
No	633(63.0)	520(89.1)	126.9	<0.001
Yes	371(36.8)	63(10.7)		
Psycological distress*				
Well (below 20)	305(39.2)	399 (65.4)	103.1	<0.001
Mild (score 20–24)	110(14,1)	68(11.1)		
Moderate (score 25–29)	50(6.4)	28(4.5)		
Severe (score 30+)	312(40.1)	115(18.8)		
Daily or almost daily tobacco use*				
No				
Yes	671(59.9)	560(77.3)		<0.001
	449(40.1)	164(22.6)	60.25	
Alcohol (AUDIT 20 or more)				
No	875(73.1)	834(97.7)		<0.001
Yes	321(26.8)	19 (2.2)	217.91	

* Totals do not add to overall total due to missing data

Similarly, there was a significant improvement in all health related components of QoL at six months except for the energy and fatigue (VT) component (Table 3). Physical and mental health scores also improved significantly.

**Table 3:** Change in quality of HRQL among TB patients after 6-months of treatment.

Variables	Baseline		Follow-up		Mean difference	
	Mean	SD	Mean	SD	Mean	p-value
General health (GH)	39.0	12.9	47.7	11.9	-8.671	<0.001
Bodily Pain (BP)	39.0	11.3	43.0	13.6	-4.043	<0.001
Physical Function (PF)	40.9	10.7	44.8	11.3	-3.838	<0.001
Physical Role (RP)	40.3	9.0	45.0	11.3	-4.715	<0.001
Social Function (SF)	39.9	12.0	44.7	13.1	-4.792	<0.001
Mental Health (MH)	41.7	8.2	43.4	8.9	-1.633	<0.001
Energy and Fatigue (Vitality) (VT)	47.6	10.7	47.8	13.6	-0.165	0.763
Emotional role (RE)	36.9	10.9	41.7	13.4	-4.794	<0.001
Physical Health Summary Score (PCS)	40.7	8.1	46.4	9.0	-5.657	<0.001
Mental Health Summary Score (MCS)	45.7	5.9	46.8	5.9	-1.182	<0.001

SD-standard deviation

### Predictors of change in physical and mental health related QoL

Although there was improvement in quality of life from baseline, however, this was only statistically significant with physical HRQL at six months follow up period (β = 1.70; p = 0.016). There was no statistically significant association between age, sex, marital status, poverty index, household income, presence of chronic conditions and daily tobacco use with both physical and mental HRQL domains after six months of treatment (Table 4). Grade 8–11 and grade 12 or more educational qualification had a strong significant positive effect on the mental health related QoL among TB patients after treatment, (β = 1.03; p = 0.041) and (β = 1.82; p = 0.008) respectively.

**Table 4:** Predictors of physical and mental health QoL among tuberculosis (TB) patients after six months of treatment.

	Physical Health Related Quality of life (HRQL)			Mental Health Related Quality of life (HRQL)		
	*β*	SE	p-value	*β*	SE	p-value
*Follow up period*						
Baseline	1			1		
Three months						
Six months	1.70	0.71	0.016	0.52	0.53	0.328
*Age in years (range 18–93)*						
18–24	1			1		
25–34	-1.47	1.04	0.158	0.59	0.77	0.441
35–44	-1.75	1.09	0.108	0.51	0.82	0.53
45–54	-1.90	1.19	0.111	1.76	0.89	0.39
55–64	-2.40	1.48	0.105	0.43	1.10	0.697
65 and above	-3.61	2.38	0.130	2.24	1.79	0.211
*Sex*						
Female	1			1		
Male	0.24	0.62	0.69	-0.35	0.46	0.446
*Marital status*						
Never married	1			1		
Married/ cohabitating	0.67	0.74	0.369	-0.02	0.56	0.971
Separated/divorced/widowed	-1.28	1.25	0.304	-0.50	0.94	0.592
*Education*						
Grade 7 or less	1			1		
Grade 8–11	-1.28	0.67	0.058	1.03	0.50	0.041
Grade 12 or more	-0.88	0.81	0.333	1.82	0.68	0.008
*Poverty index (5–20)*						
Low (5)	1			1		
Medium (6–12)	0.64	0.63	0.311	0.44	0.47	0.345
High (13–20)	1.48	0.86	0.086	-0.65	0.64	0.309
*Household income*						
Formal salary	1			1		
Family contributions	-0.06	0.72	0.924	-0.08	0.54	0.875
Social grants	-0.13	0.86	0.872	0.31	0.64	0.626
No income	1.34	1.20	0.264	0.57	0.86	0.521
*Dwelling type*						
Shack/traditional	1			1		
Permanent	0.22	0.67	0.737	1.19	0.50	0.017
**Health variables**						
*Perceived health status*						
Excellent/very good	1			1		
Good	-2.62	0.73	<0.001	0.71	0.54	0.189
Fair/poor	-10.80	0.80	<0.001	2.30	0.60	<0.001
*Chronic condition*						
No	1					
Yes	-0.27	0.70	0.695	-0.54	0.52	0.299
*Psychological distress*						
Well (below 20)	1					
Mild (score 20–24)	-4.51	0.86	<0.001	-2.12	0.64	0.001
Moderate (score 25–29)	-3.75	1.31	0.004	-4.43	0.98	<0.001
Severe (score 30+)	-5.09	0.67	<0.001	-4.97	0.50	<0.001
*Daily or almost daily tobacco use*						
No	1			1		
Yes	0.16	0.62	0.795	-0.09	0.46	0.832
*Alcohol use (AUDIT 20 or more)*						
No	1			1		
Yes	-1.74	0.79	0.028	0.21	0.59	0.725

SE-Standard error; AUDIT- Alcohol Use Disorder Identification Test (AUDIT)

**Fig 1:**
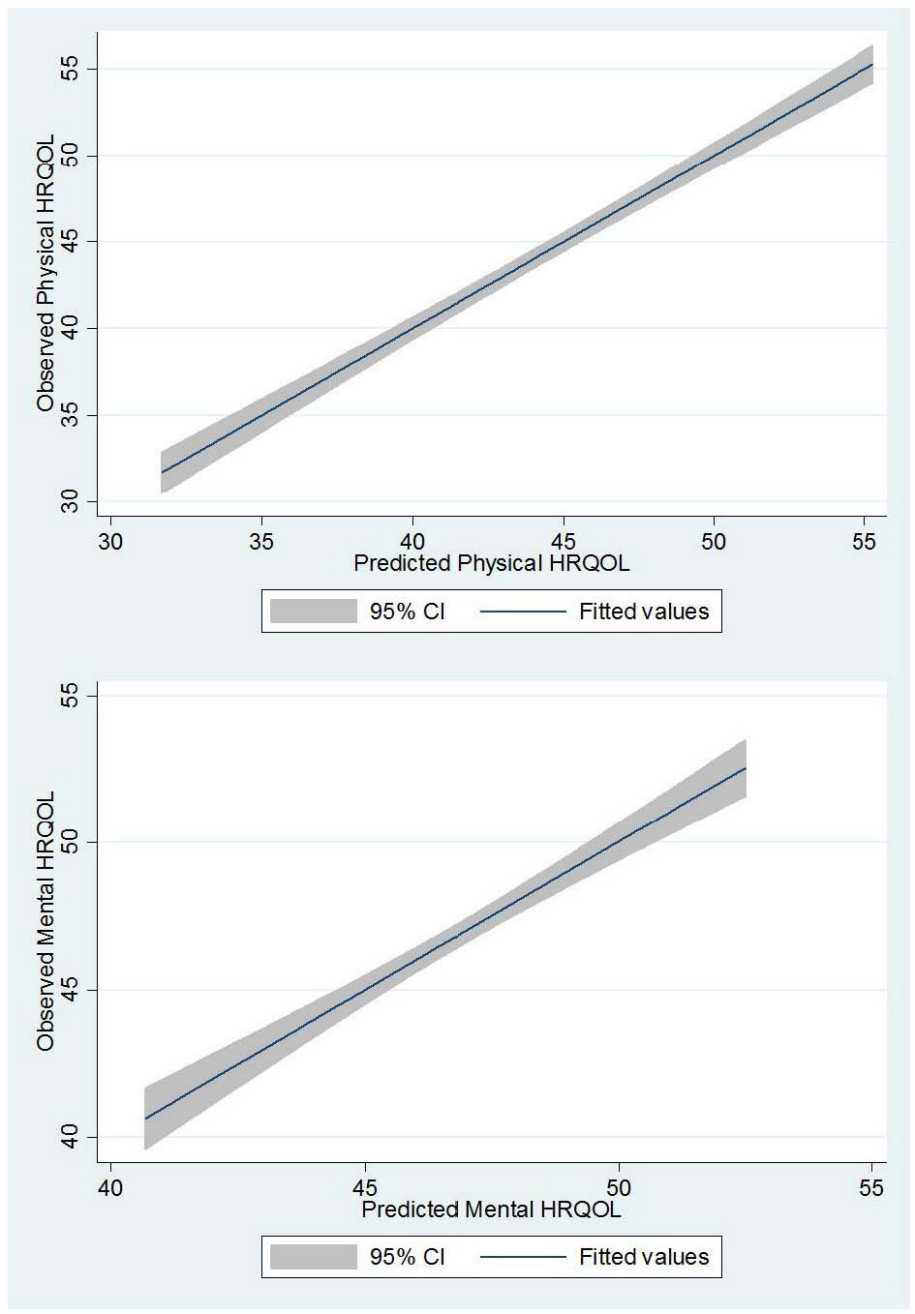
Predicted versus observed physical and mental health QoL among tuberculosis (TB) patients after six months of treatment.

Psychological distress at all levels (mild, moderate and severe) had a significant negative effect on both physical and mental HRQL after six months of treatment. Permanent residence showed a significant positive association with mental HRQL among TB patients (β = 1.19; p = 0.017) compared to those living in shack/traditional dwellings. Rating ones health as good and fair/poor was significantly associated with poor physical HRQL (β = -2.62; p < 0.001) and (β = -10.80; p < 0.001) respectively. However, rating ones health status as fair/poor was significantly associated with better mental health related quality of life (β = 2.30; p < 0.001) at 6 months. Twenty drinks or more in the past month had a significant negative effect on the physical HRQL (β = -1.74; p = 0.028). The factors independently associated with HRQL at six months after treatment were the best predictors of both physical and mental health related components of QoL (Fig 1).

## Discussion

In all patients, after 6 months of treatment there was a significant improvement in HRQL using the SF-12 instrument with the biggest increase in the physical health summary scores. In addition, each of the sub-scale scores showed significant improvement in HRQL except on the energy and fatigue (VT) component. The finding with regards to energy/ fatigue may be due to the side-effects of the anti-TB medications [24] combined with the impact of hazardous or harmful alcohol use among this particular sample. Nonetheless, various studies [25, 26, 27, 7] have reported this gradual increase across the HRQL domains after treatment using different generic HRQL instruments. But Brown et al [5] emphasized that there is still a lack of evidence regarding QoL associated with TB, its treatment and particularly the long term impact.

Our study further examined the predictors associated with change in HRQL in both the physical and mental health domains. Contrary to expectation, no statistically significant association were shown for age, sex, marital status, poverty, household income, being diagnosed with a chronic condition and daily tobacco use for either the physical or mental HRQL domain after six months. This finding is in contrast with previous studies that have identified age, being male (sex), single marital status, poverty and poor financial status as risk factors for pulmonary TB [27, 28, 29]. In particular tobacco use and co-morbid conditions such as diabetes have been reported as major drivers of the TB epidemic that is on the increase [30, 31] thus highlighting the emergence and progression of non-communicable diseases (NCD) related to TB [32]. Smoking indeed has been reported to be a predictor of poor treatment outcomes [33].

Although a recent study found no association between education and pulmonary tuberculosis when investigating risk factors for TB [29], our study found that a higher educational qualification had a strong significant positive effect on the mental HRQL among TB patients after treatment. Indeed other studies too found poorer educational status as an independent determinant of poor QoL [25, 34].

The association between alcohol use as a risk factor for TB has been well described in the literature [35, 36, 37, 38]. This study confirmed the finding, reporting harmful alcohol use in the past month having a significant negative effect on the physical HRQL. The impact of heavy drinking on the immune system can be detrimental to the physical well-being of individuals [35]. In studies elsewhere, substance abuse was the most commonly reported behavioral risk factor among patients with TB in the United States [39], the United Kingdom [40] and Russia [38]. Particularly the increased risk of TB infection related to the social interaction patterns associated with alcohol use, and in addition to the immune suppression [35] demonstrate the negative effect on the physical health of participants. However, a previous study has reported poorer QoL outcomes not only on the physical health but also on the mental health of alcohol dependent hospital outpatients in South Africa as measured by the SF-12 [41]. Most worrying though, is that patients’ improve quicker from poor physical health after treatment but their poor mental well-being tends to persist for a longer term [10, 11].

Our study shows that residing in a permanent residence has a significant positive association with mental HRQL among TB patients compared to those living in shacks/traditional dwellings. Typically in South Africa, living in a shack or traditional dwelling is associated with overcrowding and a dusty or muddy outdoor environment whereas a permanent residence is associated with less overcrowded households and a cleaner environment. Overcrowded households [42] particularly with vulnerable populations have been associated with the risk of TB infection. Rodriguez and Agbo [28] found in their study though that information on the impact on outdoor environments as risk factor of TB are scarce in South Africa. However, their study revealed that living in dusty environments increases the risk of pulmonary TB. Therefore one can deduce that exposure to better atmospheric conditions lead to better quality of life.

As expected, rating one’s health below the level of being excellent was significantly associated with poor physical HRQL. In spite of this, participants perceive their health better at 6 months and this could be due to the immediate relief in their health condition and the reduction in heavy alcohol use. However, moderate to severe psychological stress was strongly associated with poor physical and mental HRQL even after 6 months follow up. Various studies have found the presence of common mental disorders (CMDs) including anxiety and depression as a predictor of quality of life in TB patients [14, 43, 44, 45]. Particularly high levels of social anxiety with the fear of being excluded from society were found to worsen QoL outcomes for pulmonary tuberculosis patients in Turkey [46] and as a result, leading to isolation and avoidance of the topic. Diel et al. [47] noted that too often it is assumed that individuals return to normal quality of life after treatment therefore more studies are needed that focus on psychosocial risk factors including social anxiety [46] in order to gain a better understanding of what influence their circumstances and psychological health [28, 48]. Elsewhere, it has also been suggested that the screening for psychological distress, CMDs and an intervention provided could be an important strategy towards improving the QoL of patients with TB and/or HIV [14, 44].

## Study limitations

One of the biggest limitations was the lost to follow up of patients at 6-month. Furthermore, the combination of the intervention and control arms might have affected the analysis with the Screening and Brief Intervention (SBI) provided only for the treatment group, which may have impacted on the improvement in health as indicated by the SF-12 scores. Also, the self-reported improvement in health and quality of life six months after treatment is another limitation since it is prone to the possibility of recall and social desirability biases. Moreover, the study only assessed TB patients in the urban and peri-urban health facilities and therefore cannot be generalized to the rest of the South African population.

## Conclusion

Our findings suggest the importance of targeting patients with low educational background, impoverished households/ communities and hazardous or harmful alcohol use with specific interventions to improve treatment success. Social functioning (SF-12) instrument incorporating both physical and mental HRQOL scales can be used to supplement therapeutic intervention during and after treatment in order to monitor and improved clinical outcomes. However, more operational research is needed to explore how HRQL in TB can be integrated into the treatment programme.
